# Five-year prognostic significance of global longitudinal strain in individuals with a hypertrophic cardiomyopathy gene mutation without hypertrophic changes

**DOI:** 10.1007/s12471-019-1226-5

**Published:** 2019-01-24

**Authors:** H. G. van Velzen, A. F. L. Schinkel, R. W. J. van Grootel, M. A. van Slegtenhorst, J. van der Velden, M. Strachinaru, M. Michels

**Affiliations:** 1 Department of Cardiology, Thorax Centre, Erasmus Medical Centre, Rotterdam, The Netherlands; 2 Department of Clinical Genetics, Erasmus Medical Centre, Rotterdam, The Netherlands; 3 Department of Physiology, Amsterdam Cardiovascular Sciences, VU University Medical Centre, Amsterdam, The Netherlands; 4 Netherlands Heart Institute, Utrecht, The Netherlands

**Keywords:** Cardiomyopathy, Genetics, Hypertrophy, Long-term follow-up, Screening

## Abstract

**Background:**

Previous studies have reported that global longitudinal strain (GLS) is reduced in patients with hypertrophic cardiomyopathy (HCM) while left ventricular ejection fraction (LVEF) is normal. Our aim was to assess GLS in individuals with HCM mutations without hypertrophic changes and to determine its prognostic value for the development of HCM.

**Methods and results:**

This retrospective case-control and cohort study included 120 HCM mutation carriers and 110 controls. GLS and LVEF were assessed with Tomtec Imaging software. Age, gender, and body surface area were similar in mutation carriers and controls. Compared to controls, mutation carriers had a higher maximal wall thickness (9 ± 2 vs 8 ± 2 mm, *p* < 0.001), higher LVEF (60 ± 5 vs 58 ± 4%, *p* < 0.001) and higher GLS (−21.4 ± 2.3% vs −20.3 ± 2.2%, *p* < 0.001). The GLS difference was observed in the mid-left ventricle (−21.5 ± 2.5% vs −19.9 ± 2.5%, *p* < 0.001) and the apex (−24.1 ± 3.5% vs −22.1 ± 3.4%, *p* < 0.001), but not in the base of the left ventricle (−20.0 ± 3.3% vs −20.0 ± 2.6%, *p* = 0.9). Echocardiographic follow-up was performed in 80 mutation carriers. During 5.6 ± 2.9 years’ follow-up, 13 (16%) mutation carriers developed HCM. Cox regression analysis showed age (hazard ratio (HR) 1.08, *p* = 0.01), pathological Q wave (HR 8.56; *p* = 0.01), and maximal wall thickness (HR 1.94; *p* = 0.01) to be independent predictors of the development of HCM. GLS was not predictive of the development of HCM (HR 0.78, *p* = 0.07).

**Conclusion:**

GLS is increased in HCM mutation carriers without hypertrophic changes. GLS was of no clear prognostic value for the development of HCM during follow-up, in contrast to age, pathological Q waves and maximal wall thickness.

**Electronic supplementary material:**

The online version of this article (10.1007/s12471-019-1226-5) contains supplementary material, which is available to authorized users.

## What’s new?

- Global longitudinal strain is increased in hypertrophic cardiomyopathy (HCM) mutation carriers without hypertrophic changes as compared to normal individuals.
- During a 5-year follow-up of HCM mutation carriers, global longitudinal strain had no clear prognostic value for the development of HCM.
- Age, pathological Q wave, and maximal wall thickness were significant predictors of the development of HCM in HCM mutation carriers.

## Introduction

Hypertrophic cardiomyopathy (HCM) is the most common inherited cardiac disease caused by mutations in genes that encode proteins of the cardiac sarcomere [1, 2]. Genetic testing allows the identification of individuals carrying HCM mutations who do not fulfil the echocardiographic criterion of HCM [1, 2]. Due to age-related penetrance, these mutation carriers are advised to undergo periodic follow-up including electrocardiography (ECG) and echocardiography [1, 2]. Currently, we are unable to predict the development of HCM [3]. In patients with overt HCM, studies have revealed a reduced global longitudinal strain (GLS) assessed with speckle-tracking echocardiography (STE), despite a normal left ventricular ejection fraction (LVEF) [4]. Several HCM cohort studies have shown that GLS is predictive of outcome [5–7]. Data regarding GLS in HCM gene mutation carriers without hypertrophic changes are limited [8–11]. Our aim was to assess GLS in mutation carriers and healthy controls and to determine its predictive value for the development of HCM during long-term follow-up.

## Methods

This single-centre retrospective case-control and cohort study included 120 HCM mutation carriers without hypertrophic changes and 110 healthy controls, who were clinically evaluated at our cardiogenetic outpatient clinic between 2004 and 2017. For the cases, inclusion criteria were individuals with a pathogenic HCM gene mutation not fulfilling the echocardiographic criterion of HCM and with a LVEF ≥50% according to the guidelines [1, 2, 12]. Exclusion criteria were a tracking feasibility score <2 in ≥2 apical views and prior cardiovascular surgery. In total, 138 HCM mutation carriers without HCM were identified. Sixteen (12%) were excluded due to insufficient tracking feasibility and 2 (1%) because of prior cardiovascular surgery. Variants classified as class IV or V were considered pathogenic [13]. Controls were recruited via an advertisement [14]. For the controls, inclusion criteria were normal physical examination, normal ECG, and a LVEF ≥50%. Exclusion criteria were prior cardiovascular disease or risk factors, systemic disease, medication known to influence cardiac function, sports participants exercising for 6 h or more per week on a regular basis and aiming to improve their performance, body mass index >40, and women with breast implants [14]. The study conforms to the principles of the Declaration of Helsinki. Of the 138 healthy controls who were clinically evaluated, 110 (80%) were included. Ten (7%) were excluded due to insufficient tracking feasibility and 18 (13%) were randomly excluded for age and sex matching. All patients and controls gave informed consent for inclusion in the registry and local institutional review board approval was obtained.

Clinical assessment included medical history, physical examination, ECG, and transthoracic echocardiography. Studies were performed using commercially available echocardiography systems (Philips). Left ventricular hypertrophy was evaluated with the Romhilt-Estes criteria. Pathological Q waves were defined as duration >40 ms or depth >30% R wave in ≥2 leads. Maximal wall thickness, left atrial dimension, and left ventricular end-diastolic dimension were measured according to the guidelines [2, 12].

**Fig. 1:**
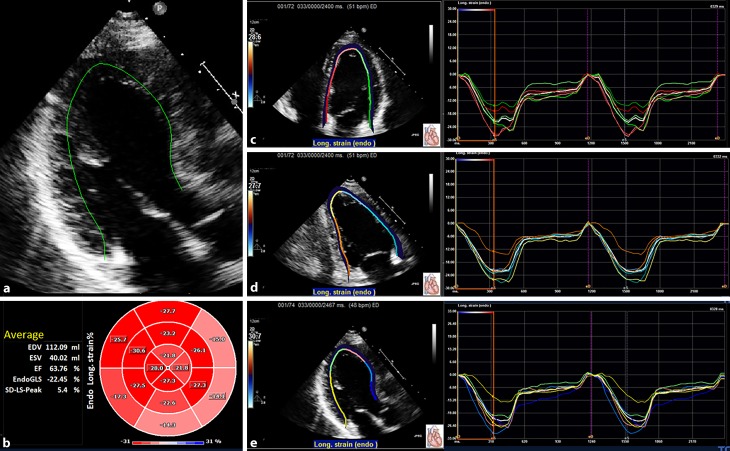
Example of left ventricular longitudinal strain measurements in a mutation carrier. The software automatically divides the left ventricular wall into 18 segments. **a** Tracing in the three-chamber view. **b** The bull’s eye in which the segmental values are plotted. On the left of the bull’s eye, the end-diastolic volume (*EDV*), end-systolic volume (*ESV*), left ventricular ejection fraction (*EF*), and global longitudinal strain (*Endo GLS*) values are presented. **c** The segmental strain curves in the four-chamber view. **d** The segmental strain curves in the two-chamber view. **e** The segmental strain curves in the three-chamber view

Standard four-chamber, two-chamber, and three-chamber views were obtained for STE analysis at frame rates of ≥50 frames/s. All STE measurements were performed by a single observer using Tomtec Imaging Systems, 2D-CPA, Build No. 1.3.0.91, Unterschleißheim, Germany. First, the cardiac cycle with the best image quality was selected. Cardiac cycles were defined by the positioning of R waves. End systole and end diastole were defined by the frame with the smallest and largest left ventricular diameter, respectively, and by determining the aortic valve closure. After manual tracing of three points designated by the software in the left ventricle on an end-systolic frame, the software automatically traced the endocardial border. Tracking feasibility in each apical view was rated with a tracking feasibility score of 3 when, on visual inspection, tracking of all myocardial segments appeared correct; 2 if it failed in one segment; and 1 if tracking was insufficient in ≥2 segments [15]. In views with foreshortening the tracking was deemed insufficient for that view. The software automatically divided the left ventricular wall into 18 segments (6 basal, 6 mid-left ventricle, and 6 apical) and calculated the longitudinal strain in all segments individually, after which the appropriate segments were averaged according to the defined region (base, mid-left ventricle, apex) (Fig. 1). Peak systolic longitudinal strain for each individual segment was defined as the peak value on the curve during the ejection phase of one cardiac cycle. The software calculated the GLS automatically. For intra-observer variability, one reader independently performed STE analysis on 20 cases in an identical fashion on two occasions with a 2-month period in between the measurements. For inter-observer variability, two readers independently performed STE analysis on 20 cases. Left ventricular end-diastolic and end-systolic volumes as well as LVEF were assessed with the biplane method of the disks using the Tomtec software.

Mortality data were obtained from the civil service register in May 2017, and were complete in 99% of the cases. Cause of death was obtained from the medical chart or the general practitioner. Echocardiographic follow-up was available in 80 mutation carriers. HCM during follow-up was defined as a maximal wall thickness ≥13 mm according to the guidelines [2].

Calculations were performed using SPSS 24 (IBM, Armonk, NY, USA). Normally distributed continuous data are expressed as mean ± standard deviation and non-normally distributed data as median followed by interquartile range. For comparing categorical variables Pearson’s chi-square test was used. For comparing continuous variables the *t*-test was used, and Mann-Whitney U in the case of non-normally distributed data. For comparison of consecutive echocardiographic data, the paired *t*-test and in the case of non-normally distributed data the Wilcoxon signed rank test were used. All analyses were two-sided; *p*-values <0.05 were considered significant. Univariate and multivariate cox regression analyses were performed and expressed as hazard ratio (HR) and 95% confidence interval (CI). Univariate significant variables were entered into the multivariable regression model.

## Results

Clinical characteristics of the study population are presented in Tab. 1. HCM gene mutation carriers represented 40 different mutations in 11 genes. The *MYBPC3* gene was most frequently affected (77%), followed by the *MYH7* gene (10%). Other genes affected were *FHL1* (3%), *TNNT2* (3%), *MYL2* (1.5%), *ALPK3* (1.5%) *MIB1* (0.8%), *MYH6* (0.8%), *TNNI3* (0.8%), *TPM1* (0.8%), and *MYL3* (0.8%).

**Table 1:** Clinical characteristics of the study population

	Mutation carriers (*n* = 120)	Controls (*n* = 110)	*p*-value
Age (years)	41 ± 13	44 ± 13	0.15
Female gender, *n* (%)	83 (69)	66 (60)	0.15
Body surface area (m^2^)	1.9 ± 0.2	1.9 ± 0.2	0.89
Systolic BP (mm Hg)	124 ± 18	125 ± 13	0.43
Diastolic BP (mm Hg)	76 ± 9	79 ± 8	0.02
Mean arterial pressure (mm Hg)	92 ± 11	94 ± 9	0.07
*Medical history*			
– Arterial hypertension, *n* (%)	9 (8)	0 (0)	0.003
– Atrial fibrillation, *n* (%)	0 (0)	0 (0)	0.50
– Diabetes mellitus, *n* (%)	2 (2)	0 (0)	0.17
– Hypercholesterolaemia, *n* (%)	1 (1)	0 (0)	0.34
*Medication*			
– Antihypertensive, *n* (%)^a^	8 (7)	0 (0)	0.01
– Statin, *n* (%)	4 (3)	0 (0)	0.05
– Antidiabetic, *n* (%)	2 (2)	0 (0)	0.17
– Antiplatelet, *n* (%)	2 (2)	0 (0)	0.17
– Oral anticoagulation, *n* (%)	1 (1)	0 (0)	0.34
*Electrocardiography*			
– Sinus rhythm, *n* (%)	120 (100)	110 (100)	0.50
– Heart rate (beats/min)	67 ± 13	59 ± 9	<0.001
– Romhilt Estes ≥4, *n* (%)	11 (9)	2 (2)	0.02
– Pathological Q wave, *n* (%)	3 (3)	0 (0)	0.10
– T-wave inversion, *n* (%)	1 (0.8)	0 (0)	0.34

Data are expressed as mean ± standard deviation or as absolute and %

^a^Includes diuretic (*n* = 3), beta-blocker (*n* = 3), ACE inhibitor (*n* = 2), angiotensin II antagonist (*n* = 2), calcium antagonist (*n* = 2)

The mean tracking feasibility score was highest in the four-chamber view (2.69 ± 1.21), followed by the three-chamber view (2.66 ± 0.58), and the two-chamber view (2.17 ± 0.74). GLS was based on measurements from three apical views in 79 (64%) mutation carriers and 86 (78%) controls (*p* = 0.02); the remaining ones were based on two apical views. Two-chamber views were most often excluded (21%) in comparison to three-chamber (6%) and four-chamber views (2%). The intra-observer agreement was 0.3 ± 1.2% and the inter-observer agreement was −0.4 ± 1.6%.

**Fig. 2:**
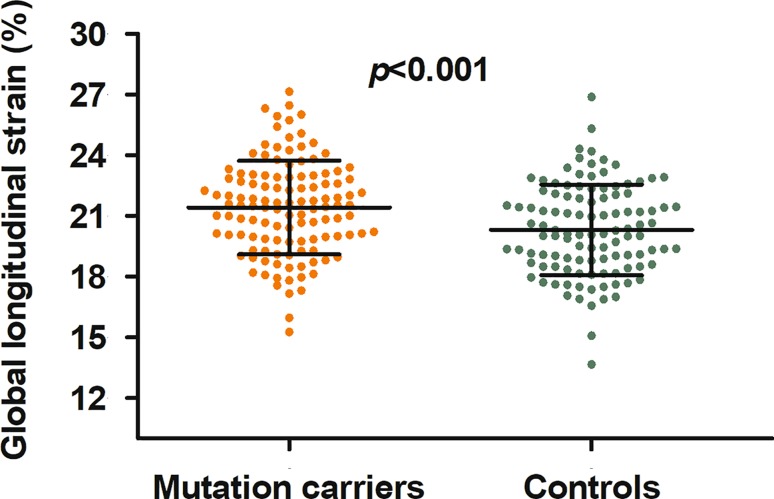
Bee swarm plot of the individual global longitudinal strain measurements in the hypertrophic cardiomyopathy mutation carriers without hypertrophic changes and in the healthy controls

Conventional echocardiographic and STE measurements are presented in Tab. 2. GLS was significantly higher in mutation carriers than in controls. When only individuals were assessed in whom all three apical views were analysable, GLS was also higher in the mutation carriers (−21.1 ± 2.3% vs −20.4 ± 2.3%, *p* = 0.04). There were significantly more mutation carriers with a GLS ≥24.7% (mean control + 2SD) than controls (8% vs 2%, *p* = 0.04). There was considerable overlap between the individual GLS measurements (Fig. 2). In both mutation carriers and controls, longitudinal strain significantly increased from the base to the apex. When compared to controls, the longitudinal strain was higher in the mid-left ventricle and apex but similar to controls in the base of the left ventricle.

**Table 2:** Findings during conventional echocardiography and speckle tracking echocardiography

	Mutation carriers (*n* = 120)	Controls (*n* = 110)	*p*-value
*Conventional echocardiography*			
Maximal wall thickness (mm)	9.4 ± 1.7	7.9 ± 1.7	<0.001
Left atrial dimension (mm)	36 ± 4	34 ± 4	0.001
Left ventricular end-diastolic dimension (mm)	46 ± 5	46 ± 4	0.39
E wave (m/s)	0.77 ± 0.17	0.71 ± 0.16	0.01
A wave (m/s)	0.57 ± 0.17	0.49 ± 0.15	<0.001
E/A ratio	1.49 ± 0.57	1.61 ± 0.67	0.16
Deceleration time (ms)	187 ± 47	189 ± 41	0.73
E’ (cm/s)	9.4 ± 2.4	9.6 ± 2.6	0.66
E/E’ ratio	8.5 ± 2.1	7.7 ± 2.0	0.004
Diastolic function, *n* (%)			
– Normal, *n* (%)	97 (89)	89 (86)	0.57
– Impaired relaxation, *n* (%)	8 (7)	5 (5)	0.45
– Pseudo normal filling, *n* (%)	4 (4)	9 (9)	0.12
– Restrictive filling, *n* (%)	0 (0)	0 (0)	0.50
*Speckle tracking echocardiography*			
Global longitudinal strain (%)	−21.4 ± 2.3	−20.3 ± 2.2	<0.001
Basal anteroseptal strain (%)	−20.1 ± 4.9	−20.3 ± 4.2	0.68
Base longitudinal strain (%)	−20.0 ± 3.3	−20.0 ± 2.6	0.87
Mid-LV longitudinal strain (%)	−21.5 ± 2.5*	−19.9 ± 2.5	<0.001
Apex longitudinal strain (%)	−24.1 ± 3.5*,**	−22.1 ± 3.4*,**	<0.001
LVEF (%)	60 ± 5	58 ± 4	<0.001
End-diastolic volume (ml)	107 ± 30	116 ± 26	0.02
End-systolic volume (ml)	42 ± 14	49 ± 13	<0.001

Data are expressed as mean ± standard deviation or absolute and %

*LV* left ventricle, *LVEF* left ventricular ejection fraction

*p < 0.05 vs base longitudinal strain, **p < 0.05 vs mid-left ventricular longitudinal strain

During 6.8 ± 3.1 (range 0.6–12.1) years’ follow-up, one (0.8%) mutation carrier died of a non-cardiac cause. Echocardiographic follow-up was performed in 80 mutation carriers (Supplementary table). During 5.6 ± 2.9 (range 1.3–11.5) years’ follow-up, 13 (16%) mutation carriers developed HCM. Those who developed HCM had a higher baseline maximal wall thickness and more ECG abnormalities (Tab. 3). GLS did not differ between the groups. In univariate Cox regression analysis, GLS was not a predictor of the development of HCM (HR 0.78; 95% CI 0.60–1.02; *p* = 0.07); neither was LVEF (HR 1.00; 95% CI 0.89–1.12; *p* = 0.95). In multivariable Cox regression analysis, age (adjusted HR 1.08, 95% CI 1.02–1.13; *p* = 0.01), pathological Q wave (adjusted HR 8.56; 95% CI 1.63–44.92; *p* = 0.01), and maximal wall thickness (HR 1.94; 95% CI 1.16–3.27; *p* = 0.01) were independent predictors of the development of HCM.

**Table 3:** Characteristics in hypertrophic cardiomyopathy mutation carriers and the occurrence of hypertrophic cardiomyopathy during follow-up

HCM during follow-up (5.6 ± 2.9 years)			
Variable	Yes (*n* = 13)	No (*n* = 67)	*p*-value
Age (years)	45 ± 18	39 ± 13	0.15
Female sex, *n* (%)	6 (46)	48 (72)	0.07
Romhilt-Estes ≥4, *n* (%)	2 (15)	5 (8)	0.36
Pathological Q wave, *n* (%)	2 (15)	1 (2)	0.02
T-wave inversion, *n* (%)	1 (8)	0 (0)	0.02
Maximal wall thickness (mm)	10.6 ± 1.4	9.3 ± 1.8	0.01
Left atrial dimension (mm)	37 ± 5	36 ± 4	0.26
Left ventricular end-diastolic diameter (mm)	46 ± 5	46 ± 5	0.89
E wave (m/s)	0.77 ± 0.16	0.77 ± 0.18	0.94
A wave (m/s)	0.57 ± 0.17	0.57 ± 0.17	0.99
E/A ratio	1.49 ± 0.52	1.48 ± 0.58	0.97
Deceleration time (ms)	176 ± 31	180 ± 46	0.81
e’ (cm/s)	9.0 ± 2.7	9.4 ± 2.5	0.62
E/e’ ratio	8.8 ± 1.7	8.5 ± 2.1	0.58
Abnormal diastolic function, *n* (%)	3 (23)	7 (11)	0.25
Global longitudinal strain (%)	−21.4 ± 2.5	−21.5 ± 2.3	0.81
Basal longitudinal strain (%)	−20.4 ± 3.0	−20.3 ± 3.5	0.93
Mid-LV longitudinal strain (%)	−21.2 ± 2.9	−21.7 ± 2.5	0.53
Apex longitudinal strain (%)	−25.4 ± 3.1	−24.0 ± 3.6	0.21
LVEF (%)	63 ± 5	60 ± 5	0.08

Data are expressed as mean ± standard deviation or absolute and %

*ECG* electrocardiography, *HCM* hypertrophic cardiomyopathy,* LV* left ventricle, *LVEF* left ventricular ejection fraction

## Discussion

The main findings of the study are: (1) GLS is increased in mutation carriers, and (2) GLS is not predictive of the development of HCM during a 5-year follow-up, in contrast to age, pathological Q wave, and maximal wall thickness.

In patients with HCM, multiple strain imaging studies have demonstrated an impaired longitudinal systolic function while LVEF is normal [4, 9, 11, 16, 17]. In mutation carriers without hypertrophy, results are contradictory. Some tissue Doppler and strain imaging studies reported lower myocardial longitudinal velocities and deformation in mutation carriers [8, 18, 19] while other studies observed no difference [9–11, 20, 21]. In the past, assessment of GLS was hampered by a lack of imaging standard. In this study, the Tomtec Imaging Systems 2D-CPA Build No. 1.3.0.91 was used, which was developed after publication of the consensus document of the EACVI/ASE/Industry Task force [22]. This allows the results to be reproducible by others and directly comparable as numbers. The current study demonstrates that GLS is increased in mutation carriers. Previously, Ho et al. similarly observed a higher GLS in *MYH7* mutation carriers [11], and De et al. reported higher tissue Doppler-derived systolic velocities implying supranormal myocardial contractility [10]. In the current study, the GLS difference between mutation carriers and controls was statistically significant. However, the clinical relevance of this difference is not sufficient in order to use GLS as a discriminating parameter, because the difference was small (~1%) and there was a large overlap of the measurements. Similar to the conclusion of Yiu et al. [9], this suggests that the assessment of GLS is not helpful for the identification of mutation carriers when genetic testing is not available.

There are multiple factors which may cause an increased GLS in HCM gene mutation carriers without hypertrophy. In line with previous studies [14, 23], we observed an increasing longitudinal strain from the base of the left ventricle towards the apex. In comparison with controls, strain was increased in the mid-left ventricle and the apex but not in the base of the left ventricle. This indicates a regional variation in the left ventricular contraction pattern. GLS may be increased as a compensatory mechanism due to subclinical dysfunction in the base of the left ventricle. Previous studies have reported a reduced septal strain in mutation carriers [9, 10]. We analysed the basal anteroseptal wall separately but found no difference between mutation carriers and controls in this region. A reduced systolic function in mutation carriers would suggest that the myocardium is diseased (i. e. coronary arteriole remodelling and muscle fibre disarray). Currently, there are no data regarding the histopathology of the myocardium in mutation carriers. However, in vivo mouse models and in vivo human studies have demonstrated a disturbance in the myocardial energy efficiency in mutation carriers without hypertrophic changes [24, 25]. Changes in myocardial efficiency may represent a primary trigger for the development of the HCM phenotype. In the future, gene-specific metabolic treatment may improve myocardial energetics and slow the progression to heart failure [26].

Another factor that might explain the increased GLS is mutation-induced cardiomyocyte hypercontractility leading to enhanced systolic function. Biophysical studies on isolated sarcomeric protein and myofilaments have demonstrated that HCM mutations increase contractility, evident from a higher actin sliding velocity, higher actomyosin ATPase activity, and increased myofilament Ca^2+^ sensitivity, resulting in a higher cardiomyocyte force at physiological [Ca^2+^] [27, 28]. A study that used myectomy samples from HCM patients harbouring sarcomere mutations demonstrated the opposite, namely a reduced force [29]. Due to the presence of cellular remodelling in tissues obtained during myectomy, it is difficult to interpret the primary consequences of the mutation. In patients with *MYH7* mutations the force generation was reduced irrespective of cellular remodelling, suggesting these mutations directly cause hypocontractility [29]. Whether HCM gene mutations cause hyper- or hypocontractility of the cardiomyocyte is subject to ongoing investigations [28]. Hypothetically, HCM mutations may initially cause hypercontractility, which then could lead to exhaustion of the cardiomyocyte in a later disease stage. Future studies exploring the temporal relation of GLS at baseline and at follow-up in mutation carriers might shed more light on this issue.

The current study is the first to evaluate the predictive value of GLS for the development of HCM, and to report no clear prognostic value during a 5-year follow-up. Nevertheless, GLS trended to be lower in subjects who developed HCM during follow-up. Future studies, preferably prospective multicentre studies with larger patient numbers, are necessary to evaluate the precise role of GLS in this specific patient cohort. A multivariate analysis demonstrated that age, pathological Q waves and maximal wall thickness were independent predictors of HCM during follow-up. The finding that advancing age is predictive supports current recommendations to perform cautionary long-term evaluation of mutation carriers without HCM [2]. The clinical utility of pathological Q waves is probably limited, because 11 out of 13 subjects who developed HCM had no pathological Q waves at initial evaluation. The association between maximal wall thickness and the development of HCM suggests extra attention to mild abnormalities indicative of HCM would be beneficial in cases with borderline wall thickness. However, the clinical significance of these mild abnormalities is probably limited [2]. This is also demonstrated by the excellent prognosis in this cohort (no cardiac deaths in 120 mutation carriers during 6.8 ± 3.1 years’ follow-up). Numerous studies have reported impaired diastolic indices in mutation carriers, suggesting diastolic dysfunction is an early phenotypic marker of HCM [9, 10, 18–21]. Ho et al. reported lower baseline E’ velocities in subjects who developed HCM during follow-up in comparison to subjects who did not develop HCM [30]. According to our data, diastolic indices including E’ had no predictive value for the development of HCM. This may be due to the fact that the mean E’ was relatively low in the whole group of mutation carriers, which is probably related to aging. In this study, 9 HCM mutation carriers had a history of arterial hypertension. Since the blood pressure was sufficiently controlled using antihypertensive medication, it is unlikely that the left ventricular wall thickening and diastolic impairment in the HCM mutation carriers were induced by arterial hypertension.

This study has several limitations. Firstly, the study population was relatively small, and echocardiographic follow-up was not performed in controls or a proportion (33%) of the mutation carriers. Secondly, GLS was based on measurements from only two apical views in a significant proportion of the subjects (36% of mutation carriers; 22% of controls), which was caused by insufficient image quality. Third, previous studies mostly used GE software, which provides mid-myocardial GLS values. The software we used only provides endocardial GLS values, limiting comparability between the studies. And finally, the majority of individuals (77%) had a mutation in the myosin-binding protein C gene; therefore results may not be applicable to carriers of other mutations.

## Conclusion

GLS is increased in HCM mutation carriers without hypertrophic changes as compared to normal individuals. GLS provided no clear prognostic value for the development of HCM during follow-up, in contrast to age, pathological Q waves and maximal wall thickness.

## Caption Electronic Supplementary Material

**Supplementary table.** Echocardiographic follow-up in 80 individuals with hypertrophic cardiomyopathy mutations
