# Supplementary material for: Five-year prognostic significance of global longitudinal strain in individuals with a hypertrophic cardiomyopathy gene mutation without hypertrophic changes
Source: Neth Heart J. 2019 Jan 24;27(3):117–26. doi: 10.1007/s12471-019-1226-5 (PMC6393574; doi:10.1007/s12471-019-1226-5)
Supplement: Supplementary file 1 — Supplementary table. Echocardiographic follow-up in 80 individuals with hypertrophic cardiomyopathy mutations [file 12471_2019_1226_MOESM1_ESM.docx]

| **Supplementary table.** Echocardiographic follow-up in 80 individuals with hypertrophic cardiomyopathy mutations. | | | |
| --- | --- | --- | --- |
| **Variable** | **Baseline** | **Follow-up** | **P-value** |
| Maximal wall thickness (mm) | 9.5±1.8 | 10.2±2.2 | 0.004 |
| Left atrial dimension (mm) | 36±5 | 35±5 | 0.15 |
| LV end-diastolic dimension (mm) | 47±5 | 45±5 | 0.01 |
| E wave (m/s) | 0.77±0.17 | 0.77±0.18 | 0.74 |
| A wave (m/s) | 0.57±0.17 | 0.64±0.19 | <0.001 |
| E/A ratio | 1.48±0.57 | 1.31±0.46 | <0.001 |
| e' (cm/s) | 9.4±2.5 | 9.1±2.5 | 0.31 |
| E/e' ratio | 8.6±2.0 | 8.8±2.2 | 0.32 |
| Deceleration time (ms) | 179±43 | 204±47 | <0.001 |
| Abnormal diastolic function, n (%) | 10 (13) | 19 (25) | 0.01 |

Data are expressed as mean ± standard deviation or absolute and %. LV = left ventricular.
